# Cost analysis of employing general practitioners within residential aged care facilities based on a prospective, stepped-wedge, cluster randomised trial

**DOI:** 10.1186/s12913-022-07766-0

**Published:** 2022-03-22

**Authors:** Lei Si, Andrew Robinson, Terry P. Haines, Petra Tierney, Andrew J. Palmer

**Affiliations:** 1 The George Institute for Global Health, UNSW Sydney, Kensington, Australia; 2 Menzies Institute for Medical Research, University of Tasmania, 17 Liverpool Street, Hobart, TAS 7000 Australia; 3 Wicking Dementia Research and Education Centre, University of Tasmania, Hobart, TAS Australia; 4 School of Primary and Allied Health Care, Monash University, Clayton, VIC Australia; 5 BUPA Aged Care Australia, Melbourne, Australia

**Keywords:** Cost analysis, Residential aged care, Stepped-wedge cluster randomised trial

## Abstract

**Objective:**

To assess the impacts of changing a model of care and employing general practitioners (GPs) within residential aged care facilities (RACFs) on costs to the aged care provider (ACP) and state and federal governments of Australia.

**Methods:**

This study was a cost analysis of a prospective, stepped-wedge, cluster randomised trial. All financial data from the ACP for every RACF involved, before and after implementation of the new model were obtained. Costs of hospital transfers, admissions, ambulance usage and GP consultations were calculated. Costs of new infrastructure, recruiting and training new staff were accounted for. Costs were standardised to 2019 Australian Dollars per occupied bed day (OBD).

**Results:**

Implementation of the new model of care resulted in overall cost savings of $9.7 per OBD to the ACP, with increased salary costs offset by increased federal government subsidies and Medicare claims income. Costs to the federal government increased by $19.6 per OBD, driven by increases in subsides. Costs savings of $3.0 per OBD to state governments were seen, driven by decreased costs of hospital transfers.

**Conclusions:**

Implementation of a model of care including GPs employed at RACFs had a mixed impact on costs depending on perspective, with overall savings to the ACP and state government perspective.

**Supplementary Information:**

The online version contains supplementary material available at 10.1186/s12913-022-07766-0.

## The known

A recent randomised controlled trial in 15 Australian residential aged care facilities (RACFs) investigated the impact of employment of general practitioners (GPs) within RACFs, appointment of a clinical manager to complement the GP, and re-allocation of registered nurse roles. The GP presence reduced the rate of unplanned hospitalisations by ~ 50%, reduced admissions to hospital and length of stay and out-of-hours calls, but increased rates of falls, infections and medication errors. The impacts on costs have not yet been investigated.

## The new

This study has shown that implementation of a model of care including GPs employed at RACFs had a mixed impact on costs depending on perspective, with overall savings to the aged care provider and state government.

## Introduction

Australia’s population, like many developed countries, is ageing rapidly, with subsequent increased demand for residential aged care facilities (RACFs). In Australia in the 2014–15 financial year, 283,268 people were in permanent residential aged care [1]. Residents in RACFs are anticipated to have different levels of acuity and care needs. The costs of aged care are rising, with the Australian Government spending increasing by 47% from 11.0 billion Australian dollars (AUD) in 2010 to AUD16.2 billion in 2015/16, of which AUD11.4 billion was spent on residential aged care [1]. High levels of frailty, comorbidities and increased levels of dependency have led to higher demand for primary medical care delivered in RACFs. The most common form of primary care delivery in RACFs in Australia consists of general practitioners (GPs) continuing to provide care for their patients as they move from a community based setting to RACFs; a model promoted by the Royal Australian College of General Practitioners (RACGPs) [2]. This model of care leads to residents being seen by GPs mainly after hours, often by locum services. A 2006 joint proposal from the Australian Medical Association and the Royal Australian College of General Practitioners identified numerous barriers and disincentives faced by GPs who care for patients RACFs, leading to fewer GPs being prepared to visit RACFs [3]. GPs on-site at RACFs may lead to reductions in potentially avoidable transfers to emergency departments [4, 5], consistent and comprehensive care with improved morale of RACF workers [6], and improved resident outcomes [7, 8].

Based on this evidence, a provider-initiated, stepped-wedge, cluster randomised controlled trial conducted over 90 weeks across 15 Australian residential aged care facilities was performed, evaluating the impact of employment of GPs as staff members within RACFs, among other models of care changes [9]. Primary outcomes were unplanned resident transfers, polypharmacy and rate of falls. GPs were recruited in only four sites, but the new model of care was implemented in all 15. The trial, including the health economics analysis, was registered on the Australia New Zealand Clinical Trial Registry in February 2013 (ACTRN12613000218796).

The new model of care had no statistically significant impact on unplanned resident transfers (incidence rate ratio (95% CI), *p*-value) 0.81 (0.66, 1.01), *p* = 0.06]; falls: 1.05 (0.94, 1.18), *p* = 0.35], or mean (SD) proportion of residents with polypharmacy 0.76 (0.09) *p* = 0.89, based on intention-to-treat analysis. While there were no clear reasons why rates of reported falls increased in the intervention period, it was conceived that unwell resident who had a higher risk of falls were retained in their aged care facilities during the intervention period, whereas they would have been transferred to the hospital or emergency department during the control period. In addition, it was noted that the RACFs residents were more vigilant and alert to recording falls when they were in the trial. Further, there were increases in reported medication errors and infections, but a reduction in hospital admissions. Post hoc analysis demonstrated that when a GP was present, the rate of unplanned hospitalisations and out of hours GP calls were halved. Reported rates of falls increased.

Remuneration problems were found as a barrier to the provision of GP services to RACFs in Australia [10]. In our new model of care, GPs were employed by a private aged care provider (ACP) which overcame the remuneration issue. However, it is crucial to understand whether the additional costs of employing GPs at RACFs can be offset by long term savings from reduced healthcare services utilisation. The cost impact of the change in the model of care with embedding of GPs at RACFs has not been explored. To address this research gap, we performed a cost analysis alongside the clinical trial of embedding in-house GPs for residential aged care facilities.

## Methods

### Trial design

**Fig. 1:**
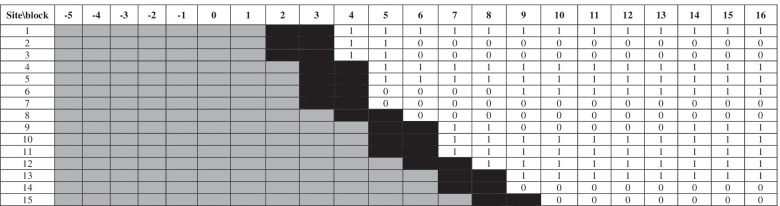
Trial design and employment of GPs at trial facilities. GP, general practitioner. Block numbers − 5 to 0 indicate retrospective period. Block numbers 1–10 indicate stepped wedge trial. Block numbers 11–16 indicate prospective follow-up period. Note that block lengths were 63 days (9 weeks), they commenced 19th December 2011, and concluded 4th October 2015. Black blocks indicate wash-out period during roll-out of the intervention. Grey blocks indicate old model of care. White blocks indicate new model of care. 0 indicates GP not employed at that site for at least half of the duration of that block. 1 indicates GP employed at that site for at least half of the duration of that block

A provider-initiated, stepped-wedge, cluster randomised controlled trial conducted over 90 weeks across 15 Australian residential aged care facilities was performed, evaluating the impact of employment of GPs as staff members within RACFs, appointment of a clinical manager to complement the GP, and re-allocation of registered nurse roles to care assistants in order to enable increased resources for resident care planning and case conferencing by registered nurses [9]. Facility GPs were employed full-time for each 150 residents at a RACF, and it was anticipated that some RACFs might share GPs because of size and geographic proximity [9]. The clinical trial ran from December 31st, 2012 to September 21st, 2014. The clinical trial design is illustrated in Fig. 1. During the trial period, there were a total of ten blocks each of 9 weeks’ duration. In addition, two blocks were assigned to each cluster as “washout” period to allow for the implementation of the new model of care in each RACF [9].

### Economic analysis

Within-trial data were used to calculate all costs of the intervention and all expenditures and income within each RACF both before and after the implementation of the new model of care. They were obtained from the Finance Department of the for-profit private ACP. The economic analysis was conducted from three perspectives: the ACP, the federal government and state government. No patient-reported outcomes were collected during the trial due to pragmatic restrictions (the trial was designed to use only data already captured in the ACP records to avoid the necessity of obtaining individual patient consent), so no analysis of cost-utility was performed. Costs were converted and expressed in 2019 Australian dollars (AUD) using the consumer price index [11].

### Costs to the ACP

Intervention costs to the ACP were calculated per occupied bed day (OBD) by the difference between the revenue and expenditure borne by the ACP. Revenue to- and expenditure by the ACP were calculated per occupied bed day (OBD) in both the before- and post-washout periods (Table 1). Expenditures were categorised by expenditures on staff, including recruitment costs and salaries for GPs.

**Table 1:** Income per occupied bed days (OBDs) breakdown for before and post-washout period, 2019 Australian dollars (AUD)

Income category (AUD)	Before-washout period^a^	Post-washout period^a^	Difference
Basic Daily Care Fees	46.8	48.6	1.8
Extra Service Fees	3.7	5.1	1.4
Accommodation Fees & Funding	19.1	19.1	0.0
Daily Accommodation Payment	–	0.3	0.3
Bond Retention Fees	3.4	3.3	−0.1
Bond Income Received	2.6	1.8	−0.8
Government Funded	–	–	–
Subsidies (including ACFI subsidies)	152.4	172.5	20.1
Payroll Tax Subsidies	8.5	8.1	−0.3
Respite Funding	3.0	2.4	−0.5
Other Subsidies	0.5	1.5	1.0
Other Income (Medicare claims, etc.)	0.7	4.1	3.4
Total income (AUD/OBD)	240.8	266.9	26.1

*ACFI* Aged Care Funding Instrument, *OBD* Occupied bed day

^a^Washout period refers to the time at the start of implementation of the intervention care model

For GP recruitment, each recruitment cost approximately AUD 7928 (as reported by the ACP), including agency and advertising fees. As there were 15 RACFs in this study and two of them shared one GP, total costs of recruiting GPs were AUD 110,995 or AUD 0.11 per OBD. The total costs of setting up a consulting room at each site were AUD 105,709. The costs of installing medication cabinets in all rooms at each RACF were AUD 21,141. Depreciation of these assets were accounted for according to the ACP accounting rules: the useful life for depreciation purposes of consultation rooms was split 80% building and 20% fittings. The AUD 84,567 relating to the building were depreciated over 50 years. The AUD 21,141 relating to fittings were depreciated at over 5 years, as were the costs of medication cabinets. Therefore, annual costs per consulting room were AUD 5920 per site. Since the average time for the post-washout period was 0.69 years, the total costs per consulting room were AUD 61,269 or AUD 0.11 per OBD. Similarly, annual costs of medication cabinets were AUD 3647. The total costs of medication cabinets were AUD 54,704 or AUD 0.11 per OBD. There were three ACP staff working on this project, the total costs for the ACP staff were AUD 951,381 or AUD 2.2 per OBD.

### Costs to government

Costs per OBD to both federal and state governments were calculated for before and post-washout periods by the summation of changes in Aged Care Funding Instrument (ACFI) subsidies [12], Medicare claims and costs of unplanned hospital transfers.

Medicare claims income for the post-washout period was recorded. As there was no specific Medicare claim data from the ACP financial department for the before-washout period (as they did not employ GPs in the pre-washout period), but there were some routine GP visits to the ACP RACFs by GPs from outside the ACP system, we have used the GP visit costs for the federal government from literature as an approximation of the costs of Medicare claims to the federal government in the before-washout period [13, 14]. It was reported that an average AUD 602 per occupied bed per year were spent on GP visit per aged care bed which is equivalent to AUD 2.1 per OBD [13, 14].

Unplanned hospital transfers were recorded by the ACP with detailed information on whether or not the patient was hospitalised following the transfer, whether the patient died in hospital, diagnosis and length of stay (LOS) in hospital. To calculate costs of unplanned hospital transfers, data from the Australian Refined Diagnosis Related Groups (AR-DRGs) Version 6.x were used [15]. Each patient transfer in this study was assigned an AR-DRG code according to the diagnosis recorded in the ACP RACF patient notes. For those who were admitted to emergency department but were discharged without inpatient care, costs were only accounted for ambulance and emergency department based on the diagnosis. Costs of ambulance service were calculated from the Australian Prudential Regulation Authority which was in accordance with the Pharmaceutical Benefits Advisory Committee (PBAC) guidelines [16, 17]. One time use of ambulance service was AUD 635. For those who died in hospital after being transferred, this cost was applied once. For those who survived and were transferred back to the residential aged care facility, it was applied twice. For those who were admitted to inpatient care, costs were calculated by the AR-DRG coding and costs were adjusted by comparing the LOS recorded in the RACF patient notes with the LOS of the corresponding AR-DRG coding [18].

### Allocation of costs to the state or federal governments

Costs of hospital transfers were separated by state and federal government. The proportion of costs paid by different levels of government was set according to hospital cost report from the Australian Institute of Health and Welfare and details of cost sharing were presented in Appendix 1 [19].

### Sensitivity analyses

In the post-washout period, different levels of GP presence were attained in each RACF, so sensitivity analyses were conducted in three subgroups: 1) RACFs with *GP present for the entire post-washout period*; 2) RACFs with *GP present at any time in post-washout period;* 3) RACFs with no *GP for the entire post-washout period*. Description of each scenario in sensitivity analyses was provided in Appendix 1. Income and expenditure to the ACP were calculated for each of the subgroups and cost of an unplanned hospital transfer was also reported in sensitivity analyses.

### Reporting quality

Reporting of the study followed the Consolidated Health Economic Evaluation Reporting Standards (CHEERS) checklist (Appendix 2) [20].

### Availability of data and materials

The datasets used and/or analysed during the current study are available from the corresponding author on reasonable request.

## Results

### Costs to the ACP

Income per OBD increased from AUD 240.8 in the before-washout period to AUD 266.9 in the post-washout period and the difference in income per OBD was AUD 26.1. Of note, the main drivers of the increase in income were ACFI subsidies and Medicare claims (Table 1). On the other hand, expenditure per OBD also increased from AUD 181.0 in the before-washout period to AUD 197.4 in the post-washout period. The difference in expenditure per OBD was AUD 16.4. The main driver of increased expenditure was staff salary (salary for GPs and nurses) (Supplementary Table 1). In total, there were cost savings of AUD 9.7 per OBD to the ACP after the implementation of the new model of care.

### Costs to government

A total of 951 unplanned hospital transfers were recorded by the ACP for the before-washout period and the average cost per hospital transfer was AUD 7279 (SD = 4683). For the post-washout period, there were a total of 994 unplanned hospital transfers with an average cost of AUD 7120 (SD = 5383) per hospital transfer. Average costs per hospital transfer decreased by AUD 37 (*p* = 0.95) for the post-washout period. In addition, for homes with a GP on site, average cost per hospital transfer decreased by AUD 542.3 (*p* = 0.13). These decreases were driven by shorter LOS for the post- versus before-washout period.

Total OBDs were 330,493 and 437,635 for before- and post-washout period respectively. Accordingly, average costs per hospital transfer per OBD were AUD 20.9 (AUD 17.0 for hospital spending and AUD 3.9 for ambulance costs) and AUD 16.2 (AUD 13.0 for hospital costs and AUD 3.2 for ambulance costs) for the before- and post-washout periods respectively, resulting in a decrease of AUD 4.8 per unplanned hospital transfer per OBD.

The cost per OBD increased by AUD 16.6 for all levels of government. The cost for unplanned hospital transfer, Medicare claims and ACFI per OBD were AUD 20.9, 2.9 and 152.4 for before washout period and AUD 16.2, 4.1 and 172.5 for post-washout period respectively. Given the cost breakdown shown in Table 2, there was a cost increase of AUD 19.6 per OBD for the federal government, driven mainly by increases in ACFI subsidies, and costs savings of AUD 3.0 per OBD for state governments, driven mainly by decreased costs of unplanned hospital transfers.

**Table 2:** Cost of medical services per occupied bed day to federal and state government for before and post-washout period, 2019 Australian dollars (AUD)

Health service	Federal government		State government	
	Before-washout	Post-washout	Before-washout	Post-washout
ACFI	152.4	172.5	0	0
Hospital	7.6	5.8	9.4	7.2
Medicare ^a^	2.9 ^b^	4.1	0	0
Ambulance	0	0	3.9	3.2

^a^Use “Other income” in Table 1 as an approximation to income from Medicare claims

^b^There were no Medicare claims from homes in before-washout period, however, it was reported that an average AUD 602.5 per occupied bed per year (or AUD 2.1 per occupied bed day) were spent on GP (not employed by The ACP) visit per aged care bed [13, 14]. Therefore, Medicare income for before-washout period was calculated by “Other income” plus AUD 2.1

Results of sensitivity analyses are presented in Appendix 1.

## Discussion

This is the first health economic analysis of employment of GPs in RACFs, either in Australia or internationally. Changing the model of care, including employment of GPs in RACFs led to increased profits to the ACP due to increases in government subsidies and Medicare payments. In addition, the new model increased costs to the Australian federal government, but saved costs to the state government due to decreased hospital transfers and length of stay if admitted.

Highest savings to the ACP seen in those homes in which a GP was present for the entire post-washout period. The lowest savings were seen in those homes in which a GP was not present at any time during the post-washout period. An intermediate effect was seen in the post-washout blocks in RACFs where GPs were present for part of the post-washout period. This effect is due to increased levels of ACFI subsidies from the federal government. GPs may assess residents at higher ACFI disability levels thereby attracting higher subsidies. Additionally, ACP income increased due to Medicare claims when the GP consulted residents. The presence of a GP in RACFs led to lower average costs per unplanned hospital transfer due to shorter length of stay and a lower likelihood of residents transferred to hospital being admitted after assessment in the emergency department. This may have resulted from hospitals being more confident to send residents back to the RACF without admission of earlier if they were admitted, knowing that a GP was there to provide potentially a higher level of post-discharge care.

Due to the complexity of hand-extraction of diagnoses related to unplanned hospital transfers, the cost analysis was only performed on data gathered from the within-trial part of the clinical study. However, 12-month pre-trial retrospective data differed little from a clinical viewpoint compared to the pre-washout period of the within trial period. The 1-year prospective post-trial follow-up period clinical results were also not substantially different to the randomised post-washout within-trial results [9], so costs were also unlikely to differ substantially in these periods.

Our study has some limitations. First, patient-reported outcomes, like multi-attribute utility or quality of life measures [21], were not collected, so a full health economic evaluation in which changes in costs were balanced against changes in these measures (in the form of cost-utility or cost-effectiveness analyses) was not possible. Second, while a detailed cost analysis in one Australian ACP setting was possible, the interpretation, application and transfer of these findings to other settings, nationally and internationally, with other health care systems and payment and subsidy arrangements should be made with caution. Third, we relied on some literature data for the cost analysis, which might not fully reflect on the individual case. For example, we used an average GP visit cost per aged care bed from a GP activity report for each individual visit. However, in reality the cost differed in each circumstance by disease complexity, time of call, and location of the ACP. Finally, this study was a within-trial cost analysis of the new model of care. The full economic benefits might not be captured within a short trial duration. Future studies are encouraged to evaluate the long-term cost-effectiveness of embedding GPs in ACPs.

## Conclusions

A change in the model of care, including employment of GPs in RACFs, had a substantial impact on incomes and expenditures, leading to an overall increased profit to the ACP, but had a differential impact on costs from state and federal government perspectives. There was an increasingly beneficial effect seen as GP presence increased, due mainly to decreased hospital transfers, decreased admissions to hospital if transferred, and decreased length of stay of admitted to hospital. Employment of GPs and changing the model of care may benefit residents as well as being profitable to ACPs, cost saving to state governments, but increased subsidy and Medicare payments may increase costs to the federal government.

## Supplementary Information

**Additional file 1.**

**Additional file 2.**

## Data Availability

The data that support the findings of this study are available from BUPA but restrictions apply to the availability of these data, which were used under license for the current study, and so are not publicly available. Data are however available from the authors upon reasonable request and with permission of BUPA.

## Abbreviations

GP: General practitioner
RACF: Residential aged care facility
ACP: Aged care provider
OBD: Occupied bed day
AUD: Australian dollars
ACFI: Aged care funding instrument
LOS: Length of stay
PBAC: Pharmaceutical Benefits Advisory Committee
CHEERS: Consolidated Health Economic Evaluation Reporting Standards
