# Supplementary material for: Cost analysis of employing general practitioners within residential aged care facilities based on a prospective, stepped-wedge, cluster randomised trial
Source: BMC Health Serv Res. 2022 Mar 22;22:374. doi: 10.1186/s12913-022-07766-0 (PMC8939179; doi:10.1186/s12913-022-07766-0)
Supplement: Supplementary file 1 — Additional file 1. [file 12913_2022_7766_MOESM1_ESM.docx]

**Appendix 1.** **Cost analysis of employing general practitioners within residential aged care facilities based on a prospective, stepped-wedge, cluster randomised trial**

**Methods**

**Costs to the aged care provider (ACP)**

Intervention costs to the ACP were calculated per occupied bed day (OBD) by the difference between the revenue and expenditure borne by the ACP. Revenue to- and expenditure by the ACP were calculated per occupied bed day (OBD) in both the before- and post-washout periods Revenues were classified by basic daily care fees, extra service fees, accommodation fees and funding, daily accommodation, bond retention fees, bond income, government funds, subsidies, payroll tax subsidies, respite funding, other subsidies and other income (Table 1). Expenditures were categorised by expenditures on staff including recruitment costs and salaries for general practitioners (GPs) and nurses, workers compensation, agency, food, incontinence aids, medical and pharmacy, domestics, premises, operations/administration, marketing and advertising, consulting, education and training, planned and unplanned repairs and maintenance, hospital service and operations replacements, diversional therapy, GP recruitment, GP consulting rooms, medication cabinets and the ACP staff time who worked on this project in the design of the intervention, interviewing candidates for the GP position, and implementation of the new model of care at each residential aged care facility (RACF) (Supplementary Table 1).

**Supplementary Table 1.** Expenditure per occupied bed days (OBDs) breakdown for before and post-washout period, 2019 Australian dollars (AUD)

| Expenditure (AUD) | Post-washout | Before-washout | Difference |
| --- | --- | --- | --- |
| Staff (GP, nurses, etc.) | 158.9 | 145.2 | 13.6 |
| Workers Compensation | 5.2 | 5.8 | -0.6 |
| Agency | 0.1 | 0.2 | -0.1 |
| Food | 8.9 | 8.6 | 0.3 |
| Incontinence Aids | 1.5 | 1.4 | 0.1 |
| Medical & Pharmacy | 2.1 | 2.1 | 0.0 |
| Domestics | 1.6 | 1.8 | -0.2 |
| Premises | 6.3 | 6.7 | -0.3 |
| Operations / Administration | 2.6 | 2.5 | 0.1 |
| Marketing & Advertising | 0.1 | 0.2 | -0.1 |
| Consulting | 0.0 | - | 0.0 |
| Education & Training | 0.6 | 0.6 | 0.0 |
| Repairs & Maintenance - Planned | 2.3 | 2.0 | 0.3 |
| Repairs & Maintenance - Unplanned | 2.5 | 1.9 | 0.6 |
| Replacements - Hospital Serv. | 0.7 | 0.7 | 0.0 |
| Replacements - Operations | 0.4 | 0.4 | 0.0 |
| Diversional Therapy | 0.6 | 0.6 | 0.0 |
| GP recruitment | 0.2 | - | 0.2 |
| GP consulting room | 0.1 | - | 0.1 |
| Medication cabinets | 0.1 | - | 0.1 |
| The ACP staff costs | 2.2 | - | 2.2 |
| Total expenditure (AUD/OBD) | 197.4 | 181.0 | 16.4 |

GP, general practitioner; OBD, occupied bed day; ACP, aged care provider.

**Costs to government**

Costs to the government of patients with hospital transfers were depended on patient destination.

For patients admitted to emergency department (ED), costs of hospital transfer were calculated as:

Cost per hospital transfer (ED admission) = C_DRG_emergency_ + C_ambulance_ $\times$ 2

where C_DRG_emergency_ denotes to cost of emergency room use by AR-DRG coding and C_ambulance_ denotes the cost of ambulance use ($\times$ 2 assuming an ambulance was used to transfer both to and from the hospital).

For patients admitted to inpatient service and *not* dying in hospital, cost of hospital transfer was calculated as:

Cost per hospital transfer (inpatient admission) = C_DRG_ + (LOS-LOS_i_) $\times$ C_hotel_fee_per_day_ +C_ambulance_$\times$2

For patients admitted to inpatient service and dying in hospital, cost of hospital transfer was calculated as:

Cost per hospital transfer (inpatient admission) = C_DRG_ + (LOS-LOS_i_) $\times$ C_hotel_fee_per_day_ +C_ambulance_

where C_DRG_ denotes the average cost of a specific AR-DRG disease, LOS-LOS_i_ denotes the difference between the actual LOS in hospital of a transferred patient and the average LOS for his or her assigned AR-DRGs coding. C_hotel_fee_per_day_ denotes the average hotel fee per day calculated from the total hotel fee divided by the average LOS for an assigned AR-DRGs coding. C_ambulance_ denotes the costs of ambulance use ($\times$ 2 assuming an ambulance was used to transfer both to and from the hospital).

**Allocation of costs to the state or federal governments**

Costs of hospital transfers were separated by state and federal government. The proportion of costs paid by different levels of government was set according to hospital cost report from the Australian Institute of Health and Welfare and details of cost sharing were presented in the Supplementary (Supplementary Table 2).(2)

**Supplementary Table 2.** Cost sharing of medical services between federal and state government

| Health service | Federal government | State governments |
| --- | --- | --- |
| ACFI | 100% | 0 |
| Hospital * | 45% | 55% |
| Medicare | 100% | 0 |
| Ambulance | 0 | 100% |

ACFI, Aged Care Funding Instrument

* Source: AIHW 2016. Australia's hospitals 2014–15 at a glance. Health services series no. 70. Cat. no. HSE 175. Canberra: AIHW. (2)

**Sensitivity analyses**

In the post-washout period, different levels of GP presence were attained in each RACF, so sensitivity analyses were conducted in three subgroups:

1. RACFs with GP present for the entire post-washout period, e.g. Site 1 from 8 July 2013to 21 September 2014 (Blocks 4-10). There were seven homes included in this subgroup: Site 1, Site 4, Site 5, Site 10, Site 11, Site 12 and Site 13 (Figure 1 in the main text).

2. RACFs with GP present at any time in post-washout period; 3). (cost calculation included the costs only in blocks in which a GP was present, as opposed to the base-case analysis that included costs for all blocks in the post-washout period whether or not a GPR was present during that block; e.g. Site2 only includes costs per OBD for 8 July-10 November 2013 (blocks 4&5 in post-black-out period). There were 11 homes included in this subgroup: Site 1-6, Site 9 and Site 10-13 (Figure 1 in the main text).

3. RACFs with no GP for the entire post-washout period, e.g. Site7 from 9 September 2013 to 21 September 2014 (Blocks 5-10). There were four homes included in this subgroup: Site 7-8, and Site 14-15 (Figure 1 in the main text).

**Results**

**Sensitivity analysis**

Income and expenditure per OBD to the ACP for post-washout period in three subgroups is given in Supplementary Table 3. Income and expenditure per OBD to the ACP for the post-washout period increased in subgroups 1 and 2 but decreased in subgroup 3 compared to the base case analysis. Further, savings to ACP also increased in subgroups 1 and 2 but decreased in subgroup 3 compared to the base case analysis.

**Supplementary Table 3.** Income and expenditure per occupied bed day to the ACP for post-washout period, 2019 Australian dollars (AUD)

|  | Income | Expenditure | Savings to ACP/OBD |
| --- | --- | --- | --- |
| **Base case:** *All RACFs* in the post-washout period included in the cost analysis | 266.9 | 197.4 | 69.6 |
| **Subgroup 1:** Only RACFs with *GP present for the* *entire post-washout period* | 277.4 | 202.2 | 75.2 |
| **Subgroup 2**: Only RACFs with *GP present at any time during post-washout period* (including those with GP present for entire period and part of the period) | 273.2 | 201.8 | 71.4 |
| **Subgroup 3:** RACFs with *no GP for the entire post-washout period* | 249.1 | 182.1 | 66.9 |

RACF, Residential aged care facility; GP, General Practitioner; ACP, aged care provider; OBD, occupied bed day.

Average and per OBD cost of an unplanned hospital transfer for post-washout period in three subgroups is summarised in Supplementary Table 4. Average and per OBD cost of an unplanned hospital transfer decreased in subgroups 1 and 2, whilst average cost increased for homes with no GP for the entire post-washout period (subgroup 3).

**Supplementary Table 4.** Average and per occupied bed day (OBD) cost of an unplanned hospital transfer for the post-washout period, 2019 Australian dollars (AUD)

|  | Average cost | Per OBD cost |
| --- | --- | --- |
| **Base case:** *All RACFs* in the post-washout period included in the cost analysis | 7120 | 16.2 |
| **Subgroup 1:** Only RACFs and with *GP present for the* *entire post-washout period* | 6911 | 14.1 |
| **Subgroup 2**: Only RACFs with *GP present at any time during post-washout period* (including those with GP present for entire period and part of the period) | 6852 | 14.6 |
| **Subgroup 3:** Only RACFs with *no GP for the entire post-washout period* | 7705 | 20.3 |

RACF = Residential aged care facility. GP = General Practitioner

**References**

1. Haines TP, Palmer AJ, Tierney P, Si L, Robinson AL. A new model of care and in-house general practitioners for residential aged care facilities: a stepped wedge, cluster randomised trial. The Medical journal of Australia. 2020.

2. Australia's hospitals 2014–15 at a glance. Canberra: AIHW; 2016.
